# Enhanced Stimulus-Induced Gamma Activity in Humans during Propofol-Induced Sedation

**DOI:** 10.1371/journal.pone.0057685

**Published:** 2013-03-06

**Authors:** Neeraj Saxena, Suresh D. Muthukumaraswamy, Ana Diukova, Krish Singh, Judith Hall, Richard Wise

**Affiliations:** 1 Department of Anaesthetics, Intensive Care and Pain Medicine, School of Medicine, Cardiff University, Cardiff, United Kingdom; 2 Department of Anaesthetics, Royal Glamorgan Hospital, Cwm Taf Local Health Board, Llantrisant, United Kingdom; 3 Cardiff University Brain Research Imaging Centre (CUBRIC), School of Psychology, Cardiff University, Cardiff, United Kingdom; University College of London - Institute of Neurology, United Kingdom

## Abstract

Stimulus-induced gamma oscillations in the 30–80 Hz range have been implicated in a wide number of functions including visual processing, memory and attention. While occipital gamma-band oscillations can be pharmacologically modified in animal preparations, pharmacological modulation of stimulus-induced visual gamma oscillations has yet to be demonstrated in non-invasive human recordings. Here, in fifteen healthy humans volunteers, we probed the effects of the GABA_A_ agonist and sedative propofol on stimulus-related gamma activity recorded with magnetoencephalography, using a simple visual grating stimulus designed to elicit gamma oscillations in the primary visual cortex. During propofol sedation as compared to the normal awake state, a significant 60% increase in stimulus-induced gamma amplitude was seen together with a 94% enhancement of stimulus-induced alpha suppression and a simultaneous reduction in the amplitude of the pattern-onset evoked response. These data demonstrate, that propofol-induced sedation is accompanied by increased stimulus-induced gamma activity providing a potential window into mechanisms of gamma-oscillation generation in humans.

## Introduction

Gamma oscillations in the 30–80 Hz range have been implicated in a wide number of functions including, memory [1], attention [2] and consciousness [3], and are thought to be disturbed in schizophrenia [4]. Both neurophysiological data and modelling studies provide convergent evidence that the most plausible mechanism for the generation of temporally-organised gamma activity is in reciprocally connected neuronal networks containing an interconnected mixture of pyramidal cells, stellate cells and GABAergic inhibitory interneurons [5], [6]. Consistent with this, gamma oscillations recorded from primary visual cortex slices *in vitro* have been shown to be modulated by drugs that target GABA_A_ receptors as well as drugs that target glutamatergic AMPA and NMDA receptors [7], and acetylcholine receptors [8]. However, the neurochemical basis and pharmacological modifiability of the spatially-summated, population-level, gamma-band responses that can be recorded from primary visual cortex non-invasively in humans with magnetoencephalography (MEG) and electroencephalography (EEG) are largely unknown.

In this experiment we attempted to modulate stimulus-induced gamma oscillations using the GABA_A_ agonist propofol. Most of the information about propofol’s in vivo modulation of neurophysiologic gamma oscillatory activity is based on investigating spontaneous EEG activity after loss of consciousness. Loss of spatiotemporal organisation of gamma oscillations and information integration capacity has been shown at anaesthetic doses of propofol [9]. However, Murphy et al [10] showed a persistently increased gamma activity with increased connectivity between the regions of the default-mode network (DMN) during propofol anaesthesia challenging the role of gamma oscillations in predicting consciousness. The relationship between spontaneous gamma activity, stimulus-induced activity and potential muscle artefacts in the spontaneous EEG is unclear [11], [12].

We investigated the modifiability of stimulus-induced gamma activity, in fifteen healthy humans during an intermediate state of consciousness, that is, sedation without loss of consciousness. MEG was used to measure oscillatory responses to a simple grating stimulus during propofol sedation and during normal wakefulness. Importantly, the stimulation paradigm and data processing techniques that we used have previously been shown to be highly reproducible, stable to repetition effects, and hence suitable for crossover neuropharmacology studies [13]. Further, MEG is robust to the muscle artefact contamination that has affected EEG studies of gamma oscillations [11], [14]. Our results demonstrate that, compared to the normal awake state, propofol-induced sedation is accompanied by an increase in visual stimulus-induced gamma-band activity as well as increased alpha desynchronisation and decreased visual evoked responses.

## Materials and Methods

### Volunteers

Fifteen right-handed, healthy, male volunteers (mean age 26 years; range 20–41 years) were recruited following a detailed screening procedure. The study was approved by Cardiff University’s Research Ethics Committee and all volunteers gave informed written consent. Medical screening was performed to ensure that all participants were in good physical and mental health and not on any regular medication (American Society of Anesthesiologists physical status 1). Any volunteer with complaints of regular heartburn or hiatus hernia, known or suspected allergies to propofol (or its constituents), regular smokers, those who snored frequently or excessively, or who had a potentially difficult-to-manage airway were excluded.

### Monitoring, Drug Administration and Sedation Assessment

Throughout the experiments, all participants were monitored in accordance with guidelines from the Association of Anaesthetists of Great Britain by two anaesthetists. Heart rate (HR), non-invasive blood pressure (BP), oxygen saturation (SpO_2_) and concentrations of expired carbon-dioxide (EtCO_2_) were continuously monitored using Veris ® MR Vital Signs monitoring system (Medrad) and recorded every 5 minutes. The monitoring system was located outside the magnetically shielded room. The connecting cables passed through waveguides into the magnetically shield room. This monitoring setup was tested and found to add no noise to the MEG signals. The monitoring anaesthetists observed the participants through a video monitor and maintained verbal contact, as required, through an intercom system.

Volunteers were instructed to follow standard pre-anaesthetic fasting guidelines. They avoided food for six hours and any fluids for two hours before the experiments. Of the two anaesthetists supervising the sessions, one was solely responsible for participant monitoring and was not actively involved in the experiment. Intravenous access (20 gauge) was obtained on the dorsum of the right hand and physiological monitoring (HR, BP, SpO_2_ and EtCO_2_) was instituted. Nasal cannulae were used for sampling of expired and inspired gases and the administration of oxygen, as required. Propofol (Propofol-Lipuro 1%, Braun Ltd., Germany) was administered using an Asena ®- PK infusion pump (Alaris Medical, UK) using a target controlled infusion based on the Marsh-pharmacokinetic model [15]. While participants lay supine in the magnetically shielded room, infusion was started targeting an effect-site concentration of 0.6 mcg/ml. Once the target was reached, two minutes were allowed to ensure reliable equilibration. Drug infusion was then increased in 0.2 mcg/ml increments until the desired level of sedation was achieved. Sedation level was assessed by an anaesthetist, blinded to the level of propofol being administered, using the modified Observer’s assessment of alertness/sedation scale (OAA/S) [16]. Sedation endpoint was an OAA/S level of 4 (slurred speech with lethargic response to verbal commands). The same anaesthetist (NS) assessed this endpoint on every occasion to ensure consistency of the depth of sedation achieved. Reaction times in response to auditory and visual stimuli were also recorded during the awake and sedated states both before and after completion of the stimulation paradigm. As expected, reaction times were significantly lower during sedation compared to waking but not significantly different before and after the stimulation session, further indicating that a steady state had been achieved.

### Stimulation Paradigm

Once steady state sedation was achieved, participants were presented with a visual stimulus consisting of a vertical, stationary, maximum contrast, three cycles per degree, square-wave grating presented on a mean luminance background. The stimulus was presented in the lower left visual field and subtended 4° both horizontally and vertically. A small red fixation square was located at the top right hand edge of the stimulus, which remained on for the entire stimulation protocol [17], [18]. The stimulus was presented on a projection screen controlled by Presentation**®**. The duration of each stimulus was 1.5–2 s followed by 2 s of fixation only. Participants were instructed to fixate for the entire experiment and in order to maintain attention were instructed to press a response key at the termination of each stimulation period. Responses slower than 750 ms triggered a brief visual warning for participants. 100 stimuli were presented in a recording session and participants responded with their right hand. Each recording session took approximately 10 min and was carried out before sedation and then repeated during sedation. The awake recording was always carried out before the sedation session on the same day. We have previously demonstrated the robustness of this paradigm to temporal order effects [13].

### MEG Acquisition and Analysis

Whole head MEG recordings were made using a CTF 275-channel radial gradiometer system sampled at 1200 Hz (0–300 Hz bandpass). An additional 29 reference channels were recorded for noise cancellation purposes and the primary sensors were analysed as synthetic third-order gradiometers [19]. Three of the 275 channels were turned off due to excessive sensor noise. At the onset of each stimulus presentation a TTL pulse was sent to the MEG system. Participants were fitted with three electromagnetic head coils (nasion and pre-auriculars), which were localised relative to the MEG system immediately before and after the recording session. Each participant had a 1 mm isotropic T_1_ weighted MRI scan available for source localisation analysis. To achieve MRI/MEG co-registration, the fiduciary markers were placed at fixed distances from anatomical landmarks identifiable in participants' anatomical MRIs (tragus, eye centre). Fiduciary locations were verified afterwards using digital photographs.

Offline, data were first epoched from −1.5 to 1.5 s around stimulus onset and each trial visually inspected for data quality. Data with gross artifacts, such as head movements and muscle contractions were excluded from further analysis. Two source localisations were performed on each dataset using synthetic aperture magnetometry, one for induced responses (SAM), and one for evoked responses (SAMerf). Correspondingly, two global covariance matrices were calculated for each dataset, one for SAM (40–80 Hz) and one for SAMerf (0–100 Hz). Based on these covariance matrices, using the beamformer algorithm [20], two sets of beamformer weights were computed for the entire brain at 4 mm isotropic voxel resolution. A multiple local-spheres [21] volume conductor model was derived by fitting spheres to the brain surface extracted by FSL's Brain Extraction Tool [22].

For gamma-band SAM imaging, virtual sensors were constructed for each beamformer voxel and student *t* images of source power changes computed using a baseline period of −1.5 to 0 s and an active period of 0 to 1.5 s. Within these images, the voxel with the strongest power increase (in the contralateral occipital lobe) was located. To reveal the time–frequency response at this peak location, the virtual sensor was repeatedly band-pass filtered between 1 and 150 Hz at 0.5 Hz frequency step intervals using an 8 Hz bandpass, 3rd order Butterworth filter [13], [23]. The Hilbert transform was used to obtain the amplitude envelope and spectra were computed as a percentage change from the mean pre-stimulus amplitude (−1.5 to 0 s) for each frequency band. This relative-change baseline provides a control for between-recording and between-participant effects (for example, different head positions in the MEG), as well as correcting for the 1/f nature of non-baseline corrected MEG source estimates [24]. From these spectra, the time courses of alpha (8–15 Hz) and gamma (40–80 Hz) were extracted and submitted to non-parametric permutation tests using 5000 permutations [25], [26]. Permuted *t* statistics were corrected for multiple comparisons using cluster-based techniques with an initial cluster forming threshold of *t* = 2.3. This approach allowed us to examine the temporal profile of oscillatory spectral modulations as well as controlling for potential contamination of early-evoked response components into the alpha band. To examine pre-stimulus amplitudes the time-frequency spectra were recomputed with no baseline correction and the average amplitudes of alpha (8–15 Hz), beta (15–40 Hz) and gamma (40–80 Hz) in the pre-stimulus period (−1.5 to 0 s) were calculated.

For SAMerf, the computed evoked response was passed through the 0–100 Hz beamformer weights and SAMerf images [27] were generated at 0.01 s intervals from 0.05 to 0.015 s. The image (usually 0.08 to 0.09 s or 0.09 to 0.1 s) with the maximal response in visual cortex was identified and the maximal voxel selected as the peak location for virtual sensor analysis. For time-domain analysis, the evoked field was computed for this virtual sensor (−0.2 to 0 s baseline, 40 Hz low-pass filter) and the peak amplitude and latency of the M100 and M150 responses were quantified. We also performed a spectral analysis of the evoked field using the same time-frequency techniques as above. The evoked frequency response in the 0 to 0.2 s period was obtained for each condition and analysed using the same statistical methodology.

## Results

**Figure 1:**
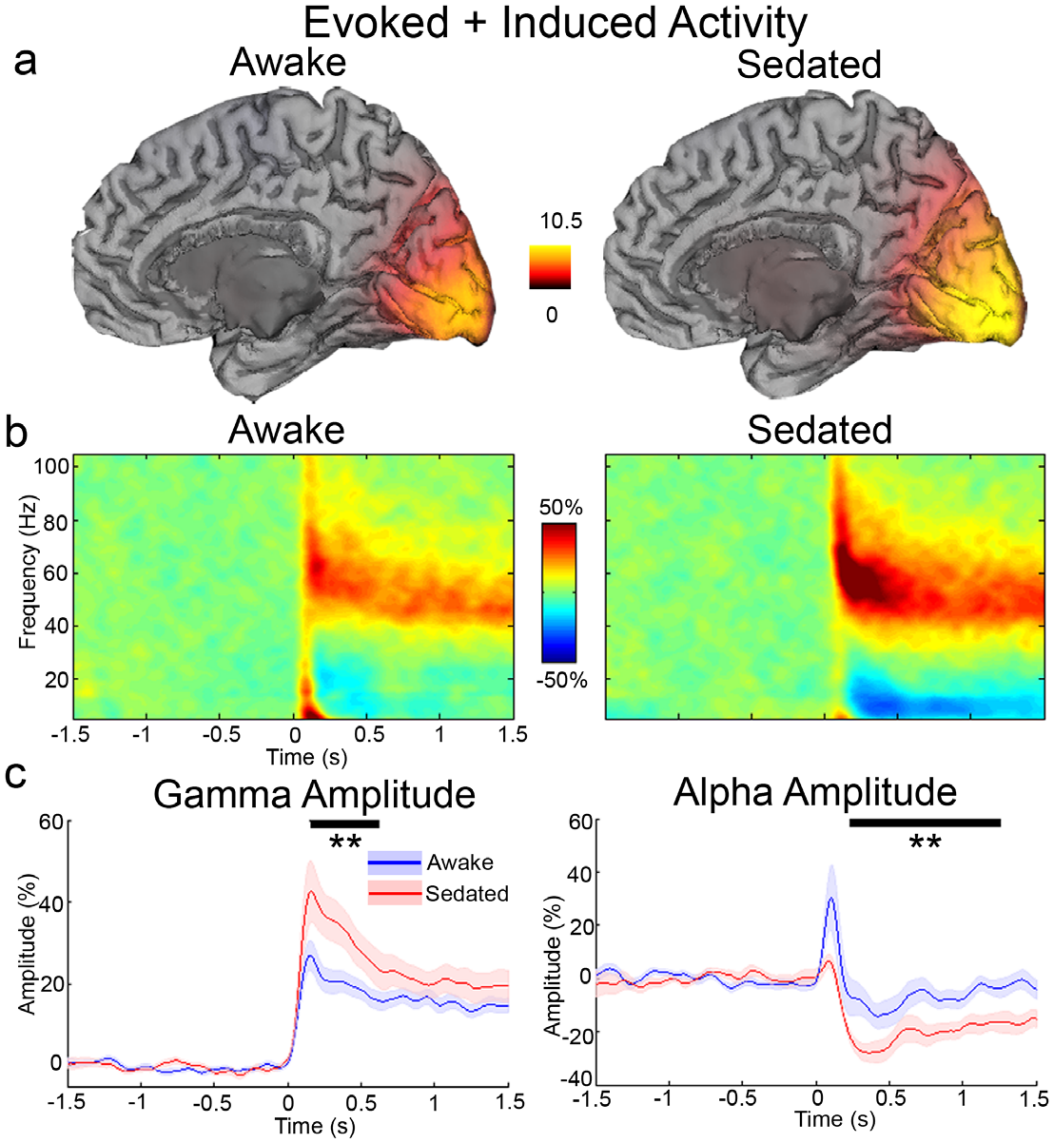
Summary of total (evoked plus induced) amplitude differences in the experiment. a) Grand-averaged source localisation of gamma oscillations (40–80 Hz) for awake and sedated states respectively. Units are *t* statistics. The peak source location for the gamma band was at MNI co-ordinate [15–95 7] for awake and [17 97 1] for sedated (adjacent SAM voxels). b) Grand-averaged time-frequency spectrograms showing source-level oscillatory amplitude (evoked+induced) changes following visual stimulation with a grating patch (stimulus onset at time = 0) during awaked and sedated states. Spectrograms are displayed as percentage change from the pre-stimulus baseline and were computed for frequencies from 5 up to 150 Hz but truncated here to 100 Hz for visualisation purposes. c) Envelopes of oscillatory amplitude for the gamma (40–80 Hz) and alpha (8–15 Hz) bands respectively. Time-periods with significant differences between the awake and sedated states are indicated with a black bar (*p<.05, **p<.01, ***p<.001, shaded areas represent SEM).

Participants showed significantly (*t* = 6.15, *p* = .001) slower key presses to stimulus offset during propofol sedation (mean 355 (s.d. 42) ms) compared to the awake state (mean 277 (33) ms). They also missed significantly more (*t* = 3.86, *p* = .002) key presses during sedation (6.1 (4.7)) compared to the awake state (1.3 (1.0)). Figure 1A shows grand-averaged source reconstructions for gamma band (40–80 Hz) responses to presentation of the grating stimulus during awake and sedated states respectively. As expected, both reconstructions locate the sources in the medial visual cortex in the quadrant opposite to the side of visual stimulation. The grand-averaged peak locations of the responses were located in adjacent source reconstruction voxels (4 mm voxel size). From the peak locations identified in individual source localisation images, source level activity was reconstructed and time-frequency spectra computed. The grand-average of these time-frequency spectra are displayed in Figure 1B. These show the typical morphology following this type of visual stimulus: there is an initial transient broadband (50 to 100 ms) amplitude increase in the gamma frequency (>40 Hz) range, followed by a longer-lasting elevation of gamma frequency amplitude in a narrower frequency range [13], [28]. In the lower frequencies, there exists a sustained alpha amplitude decrease that commences around 200 ms, and a low frequency onset response, which is indicative of the evoked response [29]. Co-localisation of alpha and gamma responses has been previously demonstrated [30]. In Figure 1C the extracted gamma (40–80 Hz) and alpha (8–15 Hz) amplitude time-courses are plotted. During propofol sedation there was significantly elevated (*p* = .01, corrected) gamma band activity between 0.15 to 0.61 s corresponding to a 59.8% increase in amplitude. Similarly, during propofol sedation there was significantly (*p*<.01, corrected) more alpha amplitude decrease between 0.230 to 1.25 s corresponding to a 94.0% increase in stimulus-induced alpha suppression.

**Figure 2:**
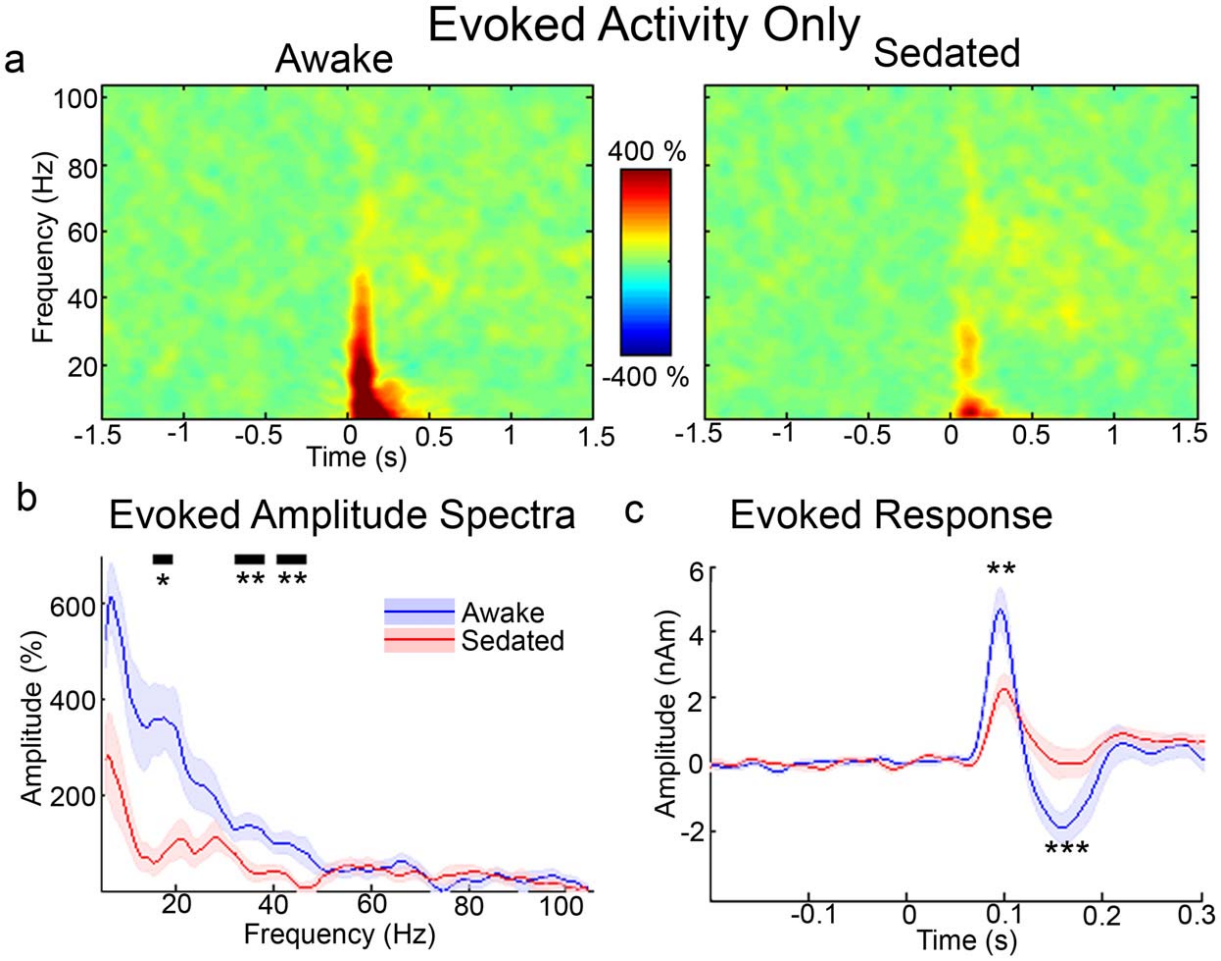
Summary of evoked amplitude differences in the experiment. a) Grand-averaged time-frequency spectrograms showing source-level oscillatory amplitude changes for the evoked response. b) Evoked amplitude spectra for the 0–0.2 s time period. c) Source-level time-averaged evoked responses for awake and sedated states. Significant differences were seen in the amplitude of the M100 and M150 responses (*p<.05, **p<.01, ***p<.001, shaded areas represent SEM).

**Figure 3:**
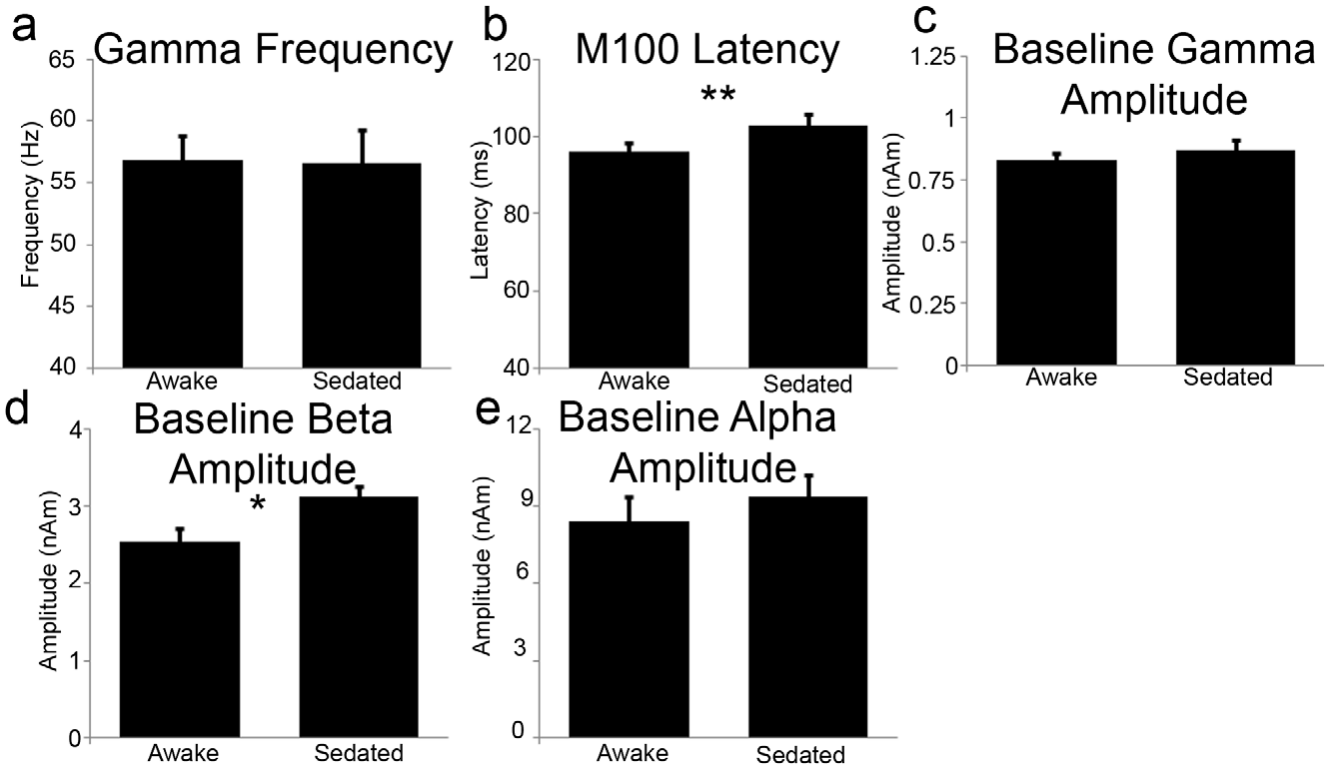
Bar charts showing peak gamma frequency (a), M100 Latency (b), and baseline gamma (c), beta (d) and alpha amplitudes (e). (*p<.05, **p<.01, ***p<.001, bars represent SEM).

In Figure 2A, the time-frequency response of the source-level evoked response is presented for both awake and sedated states and in Figure 2B the frequency spectra of these are presented for 0 to 0.2 s time window (i.e. where Figure 2A indicates that bulk of evoked activity occurred). Figure 2B indicates significantly less evoked power in the sedated state. Figure 2C presents the time-averaged evoked responses and demonstrates significant reductions in both the amplitude of the M100 (46%) and M150 (94%) components during propofol sedation. We also noted significant (*t* = 3.16, *p* = .007) slowing of the M100 component (Figure 3B). The M150 component was reduced to such a level during propofol sedation that we were unable to adequately quantify latency for a number of participants. Figure 3A demonstrates that there was no shift in peak gamma frequency, while peak alpha frequencies could not be reliably estimated across participants. We then tested whether the changes in alpha and gamma activity could be driven by changes in the baseline power spectrum. To do this, we computed the absolute amplitudes of the virtual sensor amplitude spectra in the baseline period. No changes were seen in baseline gamma or alpha amplitude but an increase in resting beta amplitude (*p* = .05) (Figure 3C–E) was seen.

We conducted exploratory correlational analyses between each of the parameters we had found to be significantly modulated by propofol (differences in, reaction time, gamma amplitude, alpha amplitude, M100 latency, M150 latency, and beta baseline amplitude). The only correlation that emerged was between M100 latency differences and alpha amplitude differences (*r* = .57, *p<*.003) and will require subsequent confirmation.

## Discussion

In this experiment, we demonstrate that during mild propofol sedation there is an increase in visually-induced gamma band responses, increased alpha amplitude suppression, and a concurrent reduction in the visually evoked response compared to the awake state. Thus, there is an overall amplification of the oscillatory response seen with visual stimulation under propofol sedation but a decrease in evoked activity. This provides an *in vivo* demonstration in humans, that stimulus-induced gamma oscillations in visual cortex can be modified pharmacologically. The increase in induced gamma and alpha stimulus reactivity occurred concurrently with a reduction in the evoked response, that is, the evoked and induced responses showed a pharmacologically-induced dissociation. One particularly striking feature of this dissociation is that this occurred in the same MEG data. This suggests that these two MEG responses may reflect the activity of different generator populations in primary visual cortex or that these generators are differentially pharmacologically sensitive. Indeed, in primary visual cortex gamma band responses are primarily generated in layers II, III and IV [31], whereas early evoked responses are mostly generated in layer IV [32]. The present dissociation appears comparable to the dissociation between ERP and the gamma responses recorded during an adaptation (double pulse paradigm) task, using subdural recordings. While there was a reduction in the ERP the gamma-band response remained constant [33]. An important aspect of this dissociation is that it argues against other, more prosaic, interpretations of the data. For example, one might argue that the reduction in the M100 amplitude evoked response is due to reduced task vigilance, attention [34] as participants’ state of consciousness changed. However, these effects would also decrease the amplitude of oscillatory responses [18], [34]. The concurrent increase in oscillatory signals is therefore inconsistent with such arguments. Another possibility is that the decreased evoked responses we observed might be due to altered fixation control during propofol sedation. However, loss of fixation control would be expected to decrease the amplitude of both the evoked response [35] and the gamma-band response [36], [18] whereas these components change in opposite directions in our data. Nevertheless, measurement of fixation position via either eye-tracking or electrooculography would be a useful addition to future experiments.

EEG studies of the resting spectra during mild propofol sedation demonstrate decreased posterior alpha and increased central beta power [37]. Increased sedation levels are marked by increased delta and theta power and frontal alpha with increased peak frequency [38]. Neural modelling of the changes in the resting EEG spectra during propofol anaesthesia suggests that these are caused by increased inhibition within local interneuron circuits [39], [40]. While the scalp EEG is a mixture of many generators, the advantage of the MEG beamformer approach used here is that it allows activity from a spatially confined region of interest to be analysed [19]. The baseline spectra in our primary visual cortex virtual sensors demonstrated only a relatively minor increase in beta power and no changes in resting gamma or alpha activity. As such, the event-related amplitude changes we demonstrate here do not appear to be related to baseline spectral changes with the drug. The other advantage of the well-validated MEG beamfomer [13], [28], [30] approach used here is that we can be very confident that the gamma-band activity here does not reflect the influence of muscle activity, be it from microsaccades [14], [41], or neck/head muscles [11].

In a recent observational study in humans we found that, across individuals, the frequency of stimulus-induced network gamma oscillations in primary visual cortex is positively correlated with the concentration of GABA measured with edited magnetic resonance spectroscopy [42]. A similar correlation between GABA concentration and gamma frequency has been observed in the motor cortex [43]. Based on these results, it might be expected that gamma frequency would increase with propofol but instead we found that gamma amplitude increased. Because, magnetic resonance spectroscopy is an indirect measure of synaptic GABA function our previous correlational results could be influenced by a number of anatomical, biochemical or even genetic variables. In particular, recently Schwarzkopf et al. [44] found across individuals, that gamma frequency correlates with the surface area of V1 defined by retinotopic mapping with fMRI, suggesting anatomical factors may have driven our previous results. While we observed here a change in gamma amplitude and not frequency, and gamma amplitude and frequency are not correlated across individuals [13], across experimental manipulations they often change together and perhaps they should not be viewed as isolated parameters. For example, in both animals [45] and humans [18], it has been shown that moving stimuli lead to gamma oscillations of both higher frequency and amplitude. Similarly, when the contrast of stimuli changes, induced gamma oscillations (dynamically) change in both amplitude [46] and frequency [47]. In addition, stimuli of different spatial frequency elicit not only different gamma amplitudes [48] but also alter the spectral shape of the gamma response [49]. Finally, recent computational modelling studies suggest that individual variability in both spatial integration across V1 columns [50] and synaptic excitation/inhibition [50], [51] can drive variability in induced visual gamma frequency, suggesting a possible dependence on multiple parameters.

While propofol exerts a small amount of activity on nAch, AMPA and NMDA receptors as well as sodium chanels its principal mechanism of action is thought to be via potentiation of GABA_A_ receptors [52]. In vitro, the primary action of propofol at low concentrations is to potentiate GABA evoked hyperpolarising Cl^-^ currents [53], [54] and at higher concentrations directly activate Cl^-^ currents via the β-subunit in human recombinant GABA_­A_ receptors [55]. At clinically relevant concentrations propofol causes a concentration dependent increase in the duration of synaptic miniature IPSCs [56], an increase in extrasynaptic tonic inhibitory currents [57] and, in hippocampal neurons, increases both the amplitude and decay time length of IPSCs [58]. Computational modelling [59] suggests that gamma activity can be generated by networks of gap junction connected interneurons [60] providing large synchronised IPSPs to excitatory cells [61]. Indeed, in barrel cortex, driving fast-spiking interneuron activity, but not pyramidal cell activity, selectively amplifies gamma activity [62]. Given all of these previous results, the amplified gamma response we observe here seems most likely to be caused by the potentiation of GABA_A_ activity by propofol. Gamma amplitude changes could result from the enhancement of either phasic or tonic GABA currents, as propofol amplifies both [63], [64], [65] and both can modify gamma activity [62], [66]. The fact that both gamma amplitude and alpha suppression were enhanced suggests an overall increase in excitatory oscillatory effects with propofol. The significant effects seen here with propofol certainly warrant future investigations using more targeted GABAergic agents.

Finally, we note another very recent study using the cholinergic agonist phystostigmine which found a selective modulation of alpha oscillation amplitude in response to visual stimuli in humans with MEG [67]. This study, which included a more attentionally demanding task than ours, also found pharmacologically altered gamma-band activity in the right frontal cortex (but not in visual cortex). Taken together, these studies demonstrate the potential of MEG to non-invasively characterise the selective effects of pharmacological agents on quantitative neuronal biomarkers.

## References

[1] Jensen O, Kaiser J, Lachaux JP (2007) Human gamma-frequency oscillations associated with attention and memory. Trends in Neurosciences 30: 317–324. 17499860 10.1016/j.tins.2007.05.001

[2] Tallon-Baudry C, Bertrand O (1999) Oscillatory gamma activity in humans and its role in object representation. Trends in Cognitive Sciences 3: 151–162. 10322469 10.1016/s1364-6613(99)01299-1

[3] Singer W (2001) Consciousness and the binding problem. Annals of the New York Academy of Science 929: 123–146. 10.1111/j.1749-6632.2001.tb05712.x 11349422

[4] Uhlhaas PJ, Singer W (2010) Abnormal neural oscillations and synchrony in schizophrenia. Nature Reviews Neuroscience 11: 100–113. 20087360 10.1038/nrn2774

[5] Bartos M, Vida I, Jonas P (2007) Synaptic mechanisms of synchronized gamma oscillations in inhibitory interneuron networks. Nature Reviews Neuroscience 8: 45–56. 17180162 10.1038/nrn2044

[6] Traub RD, Whittington MA, Colling SB, Buzsaki G, Jefferys JGR (1996) Analysis of gamma rhythms in the rat hippocampus in vitro and in vivo. Journal of Physiology-London 493: 471–484. 10.1113/jphysiol.1996.sp021397 PMC1158931 8782110

[7] Oke OO, Magony A, Anver H, Ward PD, Jiruska P, et al (2010) High-frequency gamma oscillations coexist with low-frequency gamma oscillations in the rat visual cortex in vitro. European Journal of Neuroscience 31: 1435–1445. 20384769 10.1111/j.1460-9568.2010.07171.x

[8] Rodriguez R, Kallenbach U, Singer W, Munk MH (2004) Short- and long-term effects of cholinergic modulation on gamma oscillations and response synchronization in the visual cortex. Journal of Neuroscience 24: 10369–10378. 15548651 10.1523/JNEUROSCI.1839-04.2004 PMC6730306

[9] Lee U, Mashour GA, Kim S, Noh GJ, Choi BM (2009) Propofol induction reduces the capacity for neural information integration: implications for the mechanism of consciousness and general anesthesia. Consciousness and Cognition 18: 56–64. 19054696 10.1016/j.concog.2008.10.005

[10] Murphy M, Bruno MA, Riedner BA, Boveroux P, Noirhomme Q, et al (2011) Propofol anesthesia and sleep: a high-density EEG study. Sleep 34: 283–291A. 21358845 10.1093/sleep/34.3.283 PMC3041704

[11] Whitham EM, Lewis T, Pope KJ, Fitzgibbon SP, Clark CR, et al (2008) Thinking activates EMG in scalp electrical recordings. Clinical Neurophysiology 119: 1166–1175. 18329954 10.1016/j.clinph.2008.01.024

[12] Whitham EM, Pope KJ, Fitzgibbon SP, Lewis T, Clark CR, et al (2007) Scalp electrical recording during paralysis: quantitative evidence that EEG frequencies above 20 Hz are contaminated by EMG. Clinical Neurophysiology 118: 1877–1888. 17574912 10.1016/j.clinph.2007.04.027

[13] Muthukumaraswamy SD, Singh KD, Swettenham JB, Jones DK (2010) Visual Gamma Oscillations and Evoked Responses: Variability, Repeatability and structural MRI correlates. NeuroImage 49: 3349–3357. 19944770 10.1016/j.neuroimage.2009.11.045

[14] Yuval-Greenberg S, Tomer O, Keren AS, Nelken I, Deouelll LY (2008) Transient induced gamma-band response in EEG as a manifestation of miniature saccades. Neuron 58: 429–441. 18466752 10.1016/j.neuron.2008.03.027

[15] Marsh B, White M, Morton N, Kenny GN (1991) Pharmacokinetic model driven infusion of propofol in children. British Journal of Anaesthetics 67: 41–48. 10.1093/bja/67.1.41 1859758

[16] Thomson AJ, Nimmo AF, Tiplady B, Glen JB (2009) Evaluation of a new method of assessing depth of sedation using two-choice visual reaction time testing on a mobile phone. Anaesthesia 64: 32–38. 19087003 10.1111/j.1365-2044.2008.05683.x

[17] Muthukumaraswamy SD (2010) Functional properties of human primary motor cortex gamma oscillations. Journal of Neurophysiology 104: 2873–2885. 20884762 10.1152/jn.00607.2010

[18] Swettenham JB, Muthukumaraswamy SD, Singh KD (2009) Spectral Properties of Induced and Evoked Gamma Oscillations in Human Early Visual Cortex to Moving and Stationary Stimuli. Journal of Neurophysiology 102: 1241–1253. 19515947 10.1152/jn.91044.2008

[19] Vrba J, Robinson SE (2001) Signal processing in magnetoencephalography. Methods 25: 249–271. 11812209 10.1006/meth.2001.1238

[20] Robinson SE, Vrba J (1999) Functional neuroimaging by synthetic aperture manetometry (SAM). In: Yoshimoto T, Kotani M, Kuriki S, Karibe H, Nakasato N, editors. Recent Advances in Biomagnetism. Sendai: Tohoku University Press. 302–305.

[21] Huang MX, Mosher JC, Leahy RM (1999) A sensor-weighted overlapping-sphere head model and exhaustive head model comparison for MEG. Physics in Medicine and Biology 44: 423–440. 10070792 10.1088/0031-9155/44/2/010

[22] Smith SM (2002) Fast robust automated brain extraction. Human Brain Mapping 17: 143–155. 12391568 10.1002/hbm.10062 PMC6871816

[23] Le Van Quyen M, Foucher J, Lachaux JP, Rodriguez E, Lutz A, et al (2001) Comparison of Hilbert transform and wavelet methods for the analysis of neuronal synchrony. Journal of Neuroscience Methods 111: 83–98. 11595276 10.1016/s0165-0270(01)00372-7

[24] Gross J, Baillet S, Barnes GR, Henson RN, Hillebrand A, et al (2013) Good-practice for conducting and reporting MEG research. NeuroImage 65: 349–363. 23046981 10.1016/j.neuroimage.2012.10.001 PMC3925794

[25] Maris E, Oostenveld R (2007) Nonparametric statistical testing of EEG- and MEG-data. Journal of Neuroscience Methods 164: 177–190. 17517438 10.1016/j.jneumeth.2007.03.024

[26] Nichols TE, Holmes AP (2002) Nonparametric permutation tests for functional neuroimaging: A primer with examples. Human Brain Mapping 15: 1–25. 11747097 10.1002/hbm.1058 PMC6871862

[27] Robinson SE (2004) Localization of Event-Related Activity by SAM(erf). In: Halgren E, Ahlfors S, Hamalainen M, Cohen D, editors. Boston, USA. Biomag 2004 Ltd. 16012649

[28] Hoogenboom N, Schoffelen JM, Oostenveld R, Parkes LM, Fries P (2006) Localizing human visual gamma-band activity in frequency, time and space. Neuroimage 29: 764–773. 16216533 10.1016/j.neuroimage.2005.08.043

[29] Clapp WC, Muthukumaraswamy SD, Hamm JP, Teyler TJ, Kirk IJ (2006) Long-term enhanced desynchronization of the alpha rhythm following tetanic stimulation of human visual cortex. Neuroscience Letters 398: 220–223. 16431023 10.1016/j.neulet.2005.12.081

[30] Brookes MJ, Gibson AM, Hall SD, Furlong PL, Barnes GR, et al (2005) GLM-beamformer method demonstrates stationary field, alpha ERD and gamma ERS co-localisation with fMRI BOLD response in visual cortex. Neuroimage 26: 302–308. 15862231 10.1016/j.neuroimage.2005.01.050

[31] Xing D, Yeh CI, Burns S, Shapley RM (2012) Laminar analysis of visually evoked activity in the primary visual cortex. Proceedings of the National Academy of Sciences of the United States of America 109: 13871–13876. 22872866 10.1073/pnas.1201478109 PMC3427063

[32] Kraut MA, Arezzo JC, Vaughan HG Jr (1985) Intracortical generators of the flash VEP in monkeys. Electroencephalogr Clin Neurophysiol 62: 300–312. 2408876 10.1016/0168-5597(85)90007-3

[33] Privman E, Fisch L, Neufeld MY, Kramer U, Kipervasser S, et al (2011) Antagonistic relationship between gamma power and visual evoked potentials revealed in human visual cortex. Cerebral Cortex 21: 616–624. 20624838 10.1093/cercor/bhq128

[34] Kahlbrock N, Butz M, May ES, Schnitzler A (2012) Sustained gamma band synchronization in early visual areas reflects the level of selective attention. Neuroimage 59: 673–681. 21784164 10.1016/j.neuroimage.2011.07.017

[35] Di Russo F, Martinez A, Sereno MI, Pitzalis S, Hillyard SA (2002) Cortical sources of the early components of the visual evoked potential. Human Brain Mapping 15: 95–111. 11835601 10.1002/hbm.10010 PMC6871868

[36] Perry G, Adjamian P, Thai NJ, Holliday IE, Hillebrand A, et al (2011) Retinotopic mapping of the primary visual cortex - a challenge for MEG imaging of the human cortex. European Journal of Neuroscience 34: 652–661. 21749494 10.1111/j.1460-9568.2011.07777.x PMC3178797

[37] Gugino LD, Chabot RJ, Prichep LS, John ER, Formanek V, et al (2001) Quantitative EEG changes associated with loss and return of consciousness in healthy adult volunteers anaesthetized with propofol or sevoflurane. British Journal of Anaesthesia 87: 421–428. 11517126 10.1093/bja/87.3.421

[38] Feshchenko VA, Veselis RA, Reinsel RA (2004) Propofol-induced alpha rhythm. Neuropsychobiology 50: 257–266. 15365226 10.1159/000079981

[39] Hindriks R, van Putten MJ (2002) Meanfield modeling of propofol-induced changes in spontaneous EEG rhythms. Neuroimage 60: 2323–2334. 10.1016/j.neuroimage.2012.02.042 22394672

[40] Ching S, Cimenser A, Purdon PL, Brown EN, Kopell NJ (2010) Thalamocortical model for a propofol-induced alpha-rhythm associated with loss of consciousness. Proceedings of the National Academy of Sciences of the United States of America 107: 22665–22670. 21149695 10.1073/pnas.1017069108 PMC3012501

[41] Fries P, Scheeringa R, Oostenveld R (2008) Finding gamma. Neuron 58: 303–305. 18466741 10.1016/j.neuron.2008.04.020

[42] Muthukumaraswamy SD, Edden RAE, Jones DK, Swettenham JB, Singh KD (2009) Resting GABA concentration predicts peak gamma frequency and fMRI amplitude in response to visual stimulation in humans. Proceedings of the National Academy of Sciences of the United States of America 106: 8356–8361. 19416820 10.1073/pnas.0900728106 PMC2688873

[43] Gaetz W, Edgar JC, Wang DJ, Roberts TP (2011) Relating MEG measured motor cortical oscillations to resting gamma-aminobutyric acid (GABA) concentration. Neuroimage 55: 616–621. 21215806 10.1016/j.neuroimage.2010.12.077 PMC3411117

[44] Schwarzkopf DS, Robertson DJ, Song C, Barnes GR, Rees G (2012) The frequency of visually induced gamma-band oscillations depends on the size of early human visual cortex. Journal of Neuroscience 32: 1507–1512. 22279235 10.1523/JNEUROSCI.4771-11.2012 PMC3276841

[45] Gray CM, Engel AK, Konig P, Singer W (1990) Stimulus-Dependent Neuronal Oscillations in Cat Visual Cortex: Receptive Field Properties and Feature Dependence. European Journal of Neuroscience 2: 607–619. 12106295 10.1111/j.1460-9568.1990.tb00450.x

[46] Hall SD, Holliday IE, Hillebrand A, Furlong PL, Singh KD, et al (2005) Distinct contrast response functions in striate and extra-striate regions of visual cortex revealed with magnetoencephalography (MEG). Clinical Neurophysiology 116: 1716–1722. 15953561 10.1016/j.clinph.2005.02.027

[47] Ray S, Maunsell JH (2010) Differences in gamma frequencies across visual cortex restrict their possible use in computation. Neuron 67: 885–896. 20826318 10.1016/j.neuron.2010.08.004 PMC3001273

[48] Adjamian P, Holliday IE, Barnes GR, Hillebrand A, Hadjipapas A, et al (2004) Induced visual illusions and gamma oscillations in human primary visual cortex. European Journal of Neuroscience 20: 587–592. 15233769 10.1111/j.1460-9568.2004.03495.x

[49] Hadjipapas A, Adjamian P, Swettenham JB, Holliday IE, Barnes GR (2007) Stimuli of varying spatial scale induce gamma activity with distinct temporal characteristics in human visual cortex. Neuroimage 35: 518–530. 17306988 10.1016/j.neuroimage.2007.01.002

[50] Pinotsis DA, Schwarzkopf DS, Litvak V, Rees G, Barnes G, et al (2012) Dynamic causal modelling of lateral interactions in the visual cortex. NeuroImage 66C: 563–576. 10.1016/j.neuroimage.2012.10.078 PMC3547173 23128079

[51] Chambers JD, Bethwaite B, Diamond NT, Peachey T, Abramson D, et al (2012) Parametric computation predicts a multiplicative interaction between synaptic strength parameters that control gamma oscillations. Frontiers in Computational Neuroscience 6: 53. 22837747 10.3389/fncom.2012.00053 PMC3402830

[52] Rudolph U, Antkowiak B (2004) Molecular and neuronal substrates for general anaesthetics. Nature Reviews Neuroscience 5: 709–720. 15322529 10.1038/nrn1496

[53] Concas A, Santoro G, Serra M, Sanna E, Biggio G (1991) Neurochemical action of the general anaesthetic propofol on the chloride ion channel coupled with GABAA receptors. Brain Research 542: 225–232. 1851453 10.1016/0006-8993(91)91571-h

[54] Collins GG (1988) Effects of the anaesthetic 2,6-diisopropylphenol on synaptic transmission in the rat olfactory cortex slice. British Journal of Pharmacology 95: 939–949. 2850066 10.1111/j.1476-5381.1988.tb11724.x PMC1854202

[55] Sanna E, Mascia MP, Klein RL, Whiting PJ, Biggio G, et al (1995) Actions of the general anesthetic propofol on recombinant human GABAA receptors: influence of receptor subunits. Journal of Pharmacology and Experimental Therapeutics 274: 353–360. 7616420

[56] Orser BA, Wang LY, Pennefather PS, MacDonald JF (1994) Propofol modulates activation and desensitization of GABAA receptors in cultured murine hippocampal neurons. Journal of Neuroscience 14: 7747–7760. 7996209 10.1523/JNEUROSCI.14-12-07747.1994 PMC6576906

[57] Bai D, Zhu G, Pennefather P, Jackson MF, MacDonald JF, et al (2001) Distinct functional and pharmacological properties of tonic and quantal inhibitory postsynaptic currents mediated by gamma-aminobutyric acid(A) receptors in hippocampal neurons. Molecular Pharmacology 59: 814–824. 11259626 10.1124/mol.59.4.814

[58] Whittington MA, Jefferys JG, Traub RD (1996) Effects of intravenous anaesthetic agents on fast inhibitory oscillations in the rat hippocampus in vitro. British Journal of Pharmacology 118: 1977–1986. 8864532 10.1111/j.1476-5381.1996.tb15633.x PMC1909911

[59] Wang XJ, Buzsaki G (1996) Gamma oscillation by synaptic inhibition in a hippocampal interneuronal network model. Journal of Neuroscience 16: 6402–6413. 8815919 10.1523/JNEUROSCI.16-20-06402.1996 PMC6578902

[60] Galarreta M, Hestrin S (1999) A network of fast-spiking cells in the neocortex connected by electrical synapses. Nature 402: 72–75. 10573418 10.1038/47029

[61] Hasenstaub A, Shu Y, Haider B, Kraushaar U, Duque A, et al (2005) Inhibitory postsynaptic potentials carry synchronized frequency information in active cortical networks. Neuron 47: 423–435. 16055065 10.1016/j.neuron.2005.06.016

[62] Cardin JA, Carlen M, Meletis K, Knoblich U, Zhang F, et al (2009) Driving fast-spiking cells induces gamma rhythm and controls sensory responses. Nature 459: 663–667. 19396156 10.1038/nature08002 PMC3655711

[63] Feng HJ, Macdonald RL (2004) Multiple actions of propofol on alphabetagamma and alphabetadelta GABAA receptors. Molecular Pharmacology 66: 1517–1524. 15331770 10.1124/mol.104.003426

[64] Houston CM, McGee TP, Mackenzie G, Troyano-Cuturi K, Rodriguez PM, et al (2011) Are extrasynaptic GABAA receptors important targets for sedative/hypnotic drugs? Journal of Neuroscience 32: 3887–3897. 10.1523/JNEUROSCI.5406-11.2012 PMC4620914 22423109

[65] Jeong JA, Kim EJ, Jo JY, Song JG, Lee KS, et al (2011) Major role of GABA(A)-receptor mediated tonic inhibition in propofol suppression of supraoptic magnocellular neurons. Neuroscience Letters 494: 119–123. 21376783 10.1016/j.neulet.2011.02.072

[66] Mann EO, Mody I (2011) Control of hippocampal gamma oscillation frequency by tonic inhibition and excitation of interneurons. Nature Neuroscience 13: 205–212. 10.1038/nn.2464 PMC2843436 20023655

[67] Bauer M, Kluge C, Bach D, Bradbury D, Heinze HJ, et al (2012) Cholinergic enhancement of visual attention and neural oscillations in the human brain. Current Biology 22: 397–402. 22305751 10.1016/j.cub.2012.01.022 PMC3314945

